# Management of septic joints in the United States: A Nationwide comparison of surgical and non-surgical treatment

**DOI:** 10.1007/s00264-026-06909-w

**Published:** 2026-06-24

**Authors:** Assil Mahamid, Mustafa Yassin, Basil Habiballa, Mohanad Natsheh, Hamza Murad, Dror Robinson, Barak Haviv, Ali Yassin, Muhammad Khatib

**Affiliations:** 1 Department of Orthopedics, Hasharon Hospital, Rabin Medical Center, Petah Tikva, Israel; 2 Gray Faculty of Medical & Health Sciences, Tel Aviv University, Tel Aviv, Israel; 3 Department of Orthopedics, Hillel Yaffe Medical Center, Hadera, Israel

**Keywords:** Septic arthritis, Native joint infection, Irrigation and debridement, Surgical treatment, National inpatient sample, In-hospital complications

## Abstract

**Background:**

Septic arthritis is associated with substantial morbidity and mortality. While surgical irrigation and debridement are commonly recommended, non-surgical management remains frequently utilized, particularly in medically complex patients. However, large-scale national comparisons between these strategies are limited. This study aimed to compare in-hospital outcomes of surgical versus non-surgical management using a nationwide database.

**Methods:**

A retrospective cohort study was conducted using the National Inpatient Sample (2016–2021). Adult hospitalizations with septic arthritis and known anatomic sites were identified using ICD-10 codes and stratified by treatment strategy. Primary outcomes included in-hospital mortality, length of stay, and total hospital charges. Secondary outcomes included in-hospital complications. Multivariable logistic regression was used to assess independent associations between treatment and outcomes, adjusting for demographics and comorbidities.

**Results:**

A total of 46,282 hospitalizations were identified, including 27,961 (60.4%) treated surgically and 18,321 (39.6%) non-surgically. In-hospital mortality was significantly lower in the surgical group (1.4% vs. 3.1%, p < 0.001). Length of stay was similar, though more variable in the non-surgical cohort. Surgical management was associated with higher hospital charges ($71,725 vs. $63,434, p < 0.001). Non-surgical treatment demonstrated higher rates of complications, including sepsis, acute kidney injury, respiratory failure, and thromboembolic events (all p < 0.001). After adjustment, surgical management remained independently associated with reduced complications, including respiratory failure (OR 0.552), stroke (OR 0.505), cardiac arrest (OR 0.353), and sepsis (OR 0.871) (all p < 0.001).

**Conclusions:**

Surgical management of septic arthritis was associated with lower in-hospital mortality and fewer complications despite higher costs. However, these findings should be interpreted in the context of potential residual confounding and differences in baseline illness severity between treatment groups.

**Supplementary Information:**

The online version contains supplementary material available at 10.1007/s00264-026-06909-w.

## Introduction

Septic arthritis is a bacterial infection of the joint space representing an orthopaedic and medical emergency.[1, 2] Rapid cartilage and bone destruction may occur within five to seven days, mediated by bacterial enzymes and an amplified inflammatory response.[3, 4] In-hospital mortality ranges from 2% to 15%, with one- and five-year survival rates of 76% and 54%, respectively; concurrent bacteremia independently worsens prognosis.[5–7] Up to 40% of survivors develop permanent functional impairment.[7, 8] In the United States, septic arthritis accounts for approximately 13,000–14,000 annual hospitalizations, reflecting a substantial and growing healthcare burden.[9]

The incidence is estimated at two to ten cases per 100,000 population and has increased by over 40% in recent decades.[2, 10] This rise is attributed to expanding use of joint arthroplasty, intra-articular injections, and biologic immunosuppressive therapies. High-risk populations include dialysis patients, with up to a 50-fold increased risk, and individuals with prior septic arthritis, who are at elevated risk for subsequent prosthetic joint infection.[8, 11] *Staphylococcus aureus* is the predominant pathogen, isolated in up to 54% of cases, with methicillin-resistant strains comprising 23%–48%, complicating management through biofilm formation and antimicrobial resistance.[1, 2, 4]

Management requires prompt antimicrobial therapy combined with effective joint decompression.[1, 3] However, the optimal decompression strategy remains debated. Serial needle aspiration may be appropriate in selected patients but risks inadequate source control.[12, 13] Surgical approaches, including arthroscopic lavage and open arthrotomy, provide more definitive debridement. For large joints, arthroscopy appears comparable to open techniques with lower morbidity, although higher reoperation rates have been reported in shoulder infections.[14–16] In prosthetic joint infection, surgical intervention remains essential for biofilm eradication.[17, 18] Although surgical intervention remains essential in prosthetic joint infection because of biofilm-related treatment challenges, treatment pathways for native septic arthritis differ substantially with respect to timing, operative indications, and overall management strategy. Accordingly, the present study specifically focuses on native septic arthritis requiring inpatient management.

Population-level comparative evidence between surgical and non-surgical management is limited. Existing studies are predominantly small, single-center cohorts with significant confounding, and no randomized trials have been conducted.[5–7, 12, 14] The National Inpatient Sample (NIS), a nationally representative database of US hospitalizations, enables robust large-scale evaluation of outcomes.[19]

This study aimed to characterize national epidemiology and compare outcomes of surgical versus non-surgical management of native septic arthritis across all major joint sites using NIS data from 2016–2021. We hypothesized that surgical management would be independently associated with lower in-hospital mortality and reduced complication burden.

## Methods

### Data source

A retrospective cohort study was conducted using the National Inpatient Sample (NIS) database for the years 2016–2021. The NIS is the largest publicly available all-payer inpatient database in the United States and is developed as part of the Healthcare Cost and Utilization Project (HCUP) by the Agency for Healthcare Research and Quality. It approximates a 20% stratified sample of U.S. hospital discharges and includes patient demographics, diagnoses, procedures, hospital characteristics, and in-hospital outcomes. Discharge weights provided by HCUP were applied to generate nationally representative estimates. Because the NIS database contains de-identified patient information, institutional review board approval and informed consent were not required in accordance with HCUP guidelines.

### Cohort definition and selection criteria

**Fig. 1:**
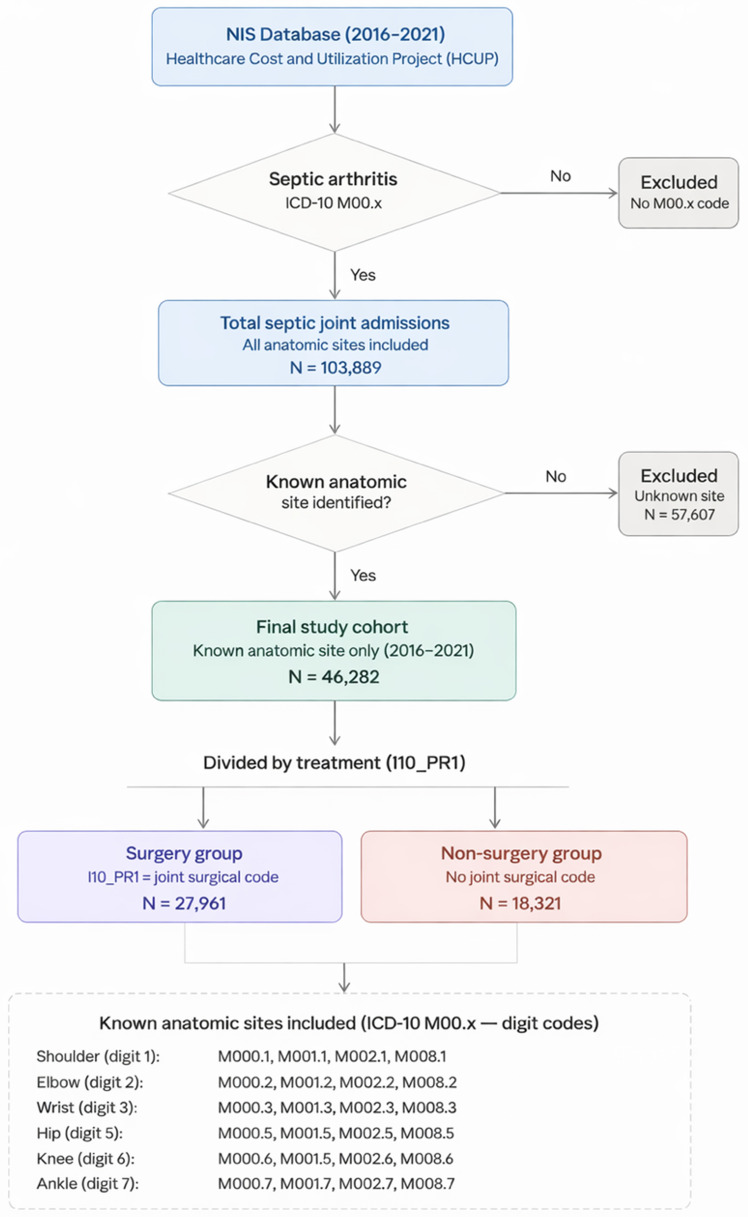
Flowchart of cohort selection and treatment group stratification for septic joint hospitalizations identified in the National Inpatient Sample (2016–2021)

Adult hospitalizations with a diagnosis of septic joint between 2016 and 2021 were identified using ICD-10-CM diagnosis codes. Only cases with known anatomic sites of septic arthritis were included in the analysis. Hospitalizations associated with prosthetic joint infection were excluded from the analysis using ICD-10 codes related to infection and inflammatory reaction due to internal joint prostheses (e.g., T84.5xx series), in order to focus specifically on native septic arthritis. This distinction was made because prosthetic joint infections differ substantially from native septic arthritis with respect to surgical management strategies, and clinical outcomes. Patients were stratified into two treatment groups based on the presence or absence of a surgical procedure. The surgical treatment group was defined by the presence of a septic joint–related procedure recorded as the primary procedure (I10_PR1) using ICD-10-PCS codes, whereas the non-surgical group included hospitalizations managed without operative intervention. The non-surgical group included hospitalizations in which no septic joint–related operative procedure code was recorded during the index admission. Because of the limitations of the NIS database, specific non-operative treatment strategies, including serial aspiration, bedside drainage, antibiotic-only management, delayed surgery after discharge, or palliative treatment approaches, could not be individually identified. Hospitalizations with missing key demographic data were excluded. The final analytic cohort consisted of 46,282 hospitalizations, including 27,961 patients treated surgically and 18,321 patients managed non-surgically. Baseline characteristics extracted from the database included patient demographics, primary payer, hospital bed size, and comorbidities. Comorbid conditions were identified using ICD-10 diagnosis codes and included common cardiovascular, metabolic, pulmonary, renal, and infectious conditions relevant to septic arthritis hospitalizations (Fig. 1).

### Missing data

Missing data were primarily related to the anatomical site of septic arthritis. From an initial cohort of 103,889 septic joint admissions identified using ICD-10 code M00.x, a substantial proportion of cases (n = 57,607) were excluded due to unspecified or unknown joint location. Consequently, only hospitalizations with clearly defined anatomic sites were retained for analysis, resulting in a final cohort of 46,282 patients. This complete-case approach was chosen to ensure anatomical homogeneity and improve the clinical interpretability of the results. Other variables included in the analysis, such as demographic characteristics, comorbidities, treatment classification, and in-hospital outcomes, had minimal missingness within the NIS database and were analyzed as recorded without imputation.

### Outcome variables (End Points)

The primary outcomes of interest were in-hospital mortality, length of stay, and total hospital charges. In-hospital mortality was defined as death during the index hospitalization. Length of stay was calculated as the number of inpatient days, and total hospital charges were reported in U.S. dollars. Secondary outcomes included in-hospital complications occurring during the admission. These complications included sepsis or septic shock, acute kidney injury, acute respiratory failure, deep vein thrombosis or pulmonary embolism, osteomyelitis, acute myocardial infarction, stroke, hospital-acquired pneumonia, hospital-acquired urinary tract infection, cardiac arrest, amputation, and wound or surgical site infection. All complications were identified using ICD-10 diagnosis and procedure codes recorded in any discharge field.

### Statistical analysis

All analyses were conducted using survey-weighted methods to account for the complex sampling design of the National Inpatient Sample, including discharge weights, stratification, and clustering. Baseline demographics, hospital characteristics, and comorbidities were compared between surgical and non-surgical groups. Categorical variables were reported as frequencies with percentages and compared using the Pearson chi-square test, while continuous variables were expressed as medians with interquartile ranges and compared using the Mann–Whitney U test due to non-normal distribution. Multivariable logistic regression models were constructed to assess the independent association between treatment strategy and in-hospital complications, adjusting for age, sex, hypertension, diabetes mellitus, obesity, chronic kidney disease, coronary artery disease, anemia, and chronic obstructive pulmonary disease. Adjusted odds ratios with 95% confidence intervals were reported. A two-sided p-value < 0.05 was considered statistically significant.

### Propensity score matching

To further reduce potential confounding by indication and address baseline differences between surgically and non-surgically managed patients, a propensity score matching (PSM) analysis was performed as a secondary analysis. Propensity scores were estimated using a multivariable logistic regression model with treatment strategy (surgical versus non-surgical management) as the dependent variable. Covariates included age, sex, race/ethnicity, primary payer, hospital bed size, hypertension, hyperlipidaemia, smoking status, diabetes mellitus, coronary artery disease, chronic kidney disease, anemia, electrolyte disorders, hypothyroidism, chronic obstructive pulmonary disease, obesity, chronic pain disorders, anxiety disorders, cellulitis, bacteraemia, sepsis, gastroesophageal reflux disease, benign prostatic hyperplasia, osteoarthritis, and urinary tract infection. Patients were matched in a 1:1 ratio using nearest-neighbour matching without replacement. Covariate balance after matching was assessed using standardized mean differences (SMDs), with values <0.10 considered indicative of adequate balance. Outcomes were subsequently compared between matched cohorts using appropriate univariate statistical tests.

### Ethics and institutional review board approval

This study utilized the National Inpatient Sample (NIS), a publicly available, de-identified database developed as part of the Healthcare Cost and Utilization Project (HCUP). As the dataset does not contain direct patient identifiers and complies with the Health Insurance Portability and Accountability Act (HIPAA), this study did not involve human subjects as defined by federal regulations. Accordingly, institutional review board (IRB) approval and informed consent were not required. The study was conducted in accordance with HCUP data use agreements and relevant ethical guidelines for research using de-identified administrative data.

## Results

Panel A illustrates the annual number of septic arthritis cases across all joint sites, including knee, hip, ankle, and cases with unspecified location, along with the overall trend. The knee represented the most frequently involved joint throughout the study period, followed by the hip and ankle. Cases categorized as unknown location comprised a substantial proportion of the total burden. Overall, the number of cases increased steadily from 2016 to 2019, followed by a decline in 2020–2021.

**Fig. 2:**
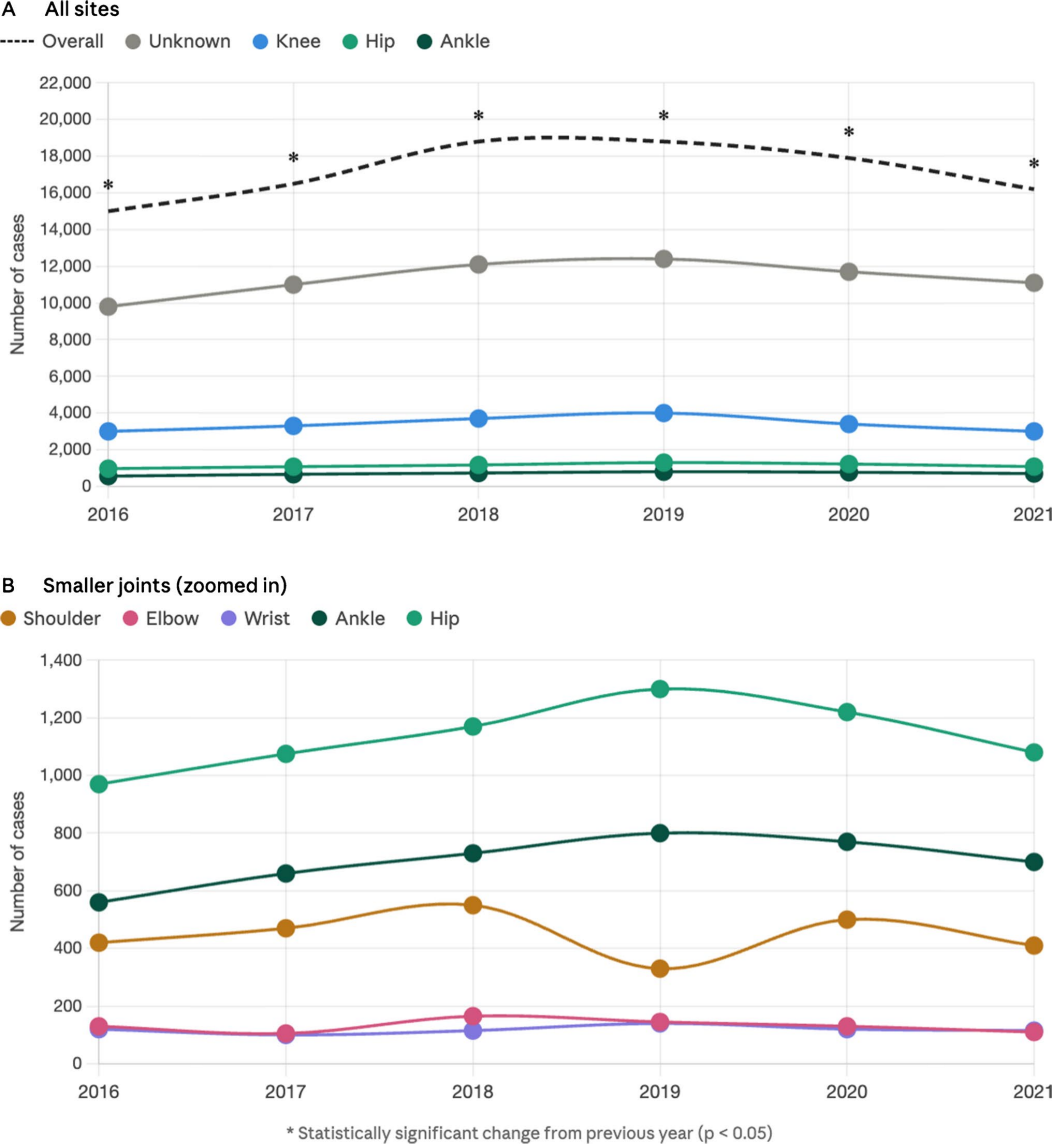
Temporal Trends in Septic Arthritis Cases by Joint Location in the United States, 2016–2021

Panel B provides a zoomed-in view of smaller joints, including shoulder, elbow, and wrist. Among these, the shoulder was the most commonly affected small joint, whereas elbow and wrist involvement remained relatively infrequent and stable over time. Similar to the overall trend, smaller joint infections demonstrated a gradual increase until 2019 with a subsequent decrease in the later years (Fig. 2).

A total of 46,282 hospitalizations for septic arthritis with known anatomic sites were included, of which 27,961 (60.4%) underwent surgical management and 18,321 (39.6%) were treated non-surgically. The cohort was predominantly male (63.4%), with no significant difference between groups (p = 0.887). Race and ethnicity distributions differed modestly (p = 0.030), with White patients comprising the majority (70.0%), followed by Black (12.5%) and Hispanic patients (9.0%), with only minor intergroup variation. Primary payer status differed significantly (p < 0.001): Medicare was most common (46.6%) and similarly distributed, whereas Medicaid coverage was more frequent in the non-surgical group (22.7% vs. 19.2%), and private insurance was more common among surgically treated patients (25.8% vs. 21.4%). Most admissions occurred in large hospitals (52.1%), with smaller proportions in medium (27.0%) and small centers (21.0%), showing modest but significant differences between groups (p = 0.031). Overall, demographic and institutional differences suggest potential disparities in treatment allocation (Table 1).

**Table 1:** Baseline demographic and hospital characteristics of patients with septic joint admissions stratified by surgical versus non-surgical management (NIS 2016–2021)

Characteristic	Overall (N = 46,282)	Surgery (n = 27,961)	No Surgery (n = 18,321)	*p*-value
Sex, n (%)				**0.887**
Male	29,356 (63.4%)	17,743 (63.5%)	11,613 (63.4%)	0.887
Female	16,916 (36.5%)	10,212 (36.5%)	6,704 (36.6%)	0.887
Race/Ethnicity, n (%)				**0.030**
White	32,401 (70.0%)	19,711 (70.5%)	12,690 (69.3%)	0.030
Black	5,763 (12.5%)	3,421 (12.2%)	2,342 (12.8%)	0.030
Hispanic	4,176 (9.0%)	2,535 (9.1%)	1,641 (9.0%)	0.030
Asian/Pacific Islander	780 (1.7%)	470 (1.7%)	310 (1.7%)	0.030
Native American	627 (1.4%)	353 (1.3%)	274 (1.5%)	0.030
Other	949 (2.1%)	548 (2.0%)	401 (2.2%)	0.030
Primary Payer, n (%)				**<0.001**
Medicare	21,562 (46.6%)	13,019 (46.6%)	8,543 (46.6%)	<0.001
Medicaid	9,515 (20.6%)	5,364 (19.2%)	4,151 (22.7%)	<0.001
Private Insurance	11,131 (24.1%)	7,206 (25.8%)	3,925 (21.4%)	<0.001
Self-Pay	2,163 (4.7%)	1,215 (4.3%)	948 (5.2%)	<0.001
No Charge	209 (0.5%)	122 (0.4%)	87 (0.5%)	<0.001
Other	1,626 (3.5%)	996 (3.6%)	630 (3.4%)	<0.001
Hospital Bed Size, n (%)				**0.031**
Small	9,715 (21.0%)	5,932 (21.2%)	3,783 (20.6%)	0.031
Medium	12,476 (27.0%)	7,613 (27.2%)	4,863 (26.5%)	0.031
Large	24,091 (52.1%)	14,416 (51.6%)	9,675 (52.8%)	0.031

Data are presented as n (%). Categorical variables compared using Pearson chi-square test. NIS = National Inpatient Sample. Surgery defined by ICD-10-PCS primary procedure code (I10_PR1). p < 0.05 considered statistically significant.

Baseline comorbidities stratified by treatment group are summarized in Table 2. Hypertension was the most prevalent condition (60.7%) and was slightly more common in the surgical group (61.4% vs. 59.5%, p < 0.001), as was hyperlipidaemia (34.1% vs. 30.5%, p < 0.001). Diabetes mellitus was common (36.1%) without intergroup difference (p = 0.553). Several comorbidities were more frequent in the non-surgical cohort, including coronary artery disease (18.5% vs. 16.5%), chronic kidney disease (36.6% vs. 31.7%), anaemia (45.8% vs. 42.8%), electrolyte disorders (37.9% vs. 34.1%), COPD (18.0% vs. 17.0%), and anxiety disorders (17.0% vs. 15.7%) (all p ≤ 0.009). Infectious conditions, including cellulitis (33.8% vs. 27.5%), sepsis (31.5% vs. 28.3%), and urinary tract infection (9.6% vs. 7.9%), were also more common in non-surgically managed patients (all p < 0.001). Conversely, obesity (24.1% vs. 20.4%), osteoarthritis (17.8% vs. 12.6%), and gastroesophageal reflux disease (20.2% vs. 18.4%) were more prevalent in the surgical group (all p < 0.001). Bacteraemia was highly prevalent (79.5%) with minimal intergroup difference. Overall, non-surgical patients demonstrated a higher systemic comorbidity and infectious burden.

**Table 2:** Baseline comorbidities of patients with septic joint admissions stratified by surgical versus non-surgical management (NIS 2016–2021)

Comorbidity	Overall (N = 46,282)	Surgery (n = 27,961)	No Surgery (n = 18,321)	*p*-value
Hypertension	28,074 (60.7%)	17,174 (61.4%)	10,900 (59.5%)	<0.001
Hyperlipidemia	15,116 (32.7%)	9,530 (34.1%)	5,586 (30.5%)	<0.001
Smoking/Nicotine use	17,879 (38.6%)	10,647 (38.1%)	7,232 (39.5%)	0.003
Diabetes mellitus	16,717 (36.1%)	10,069 (36.0%)	6,648 (36.3%)	0.553
Coronary artery disease	8,011 (17.3%)	4,624 (16.5%)	3,387 (18.5%)	<0.001
Chronic kidney disease/AKI	15,571 (33.6%)	8,867 (31.7%)	6,704 (36.6%)	<0.001
Anemia (all types)	20,350 (44.0%)	11,957 (42.8%)	8,393 (45.8%)	<0.001
Electrolyte disorders	16,478 (35.6%)	9,541 (34.1%)	6,937 (37.9%)	<0.001
Hypothyroidism	5,055 (10.9%)	3,089 (11.0%)	1,966 (10.7%)	0.292
COPD	8,053 (17.4%)	4,760 (17.0%)	3,293 (18.0%)	0.009
Obesity/Morbid obesity	10,478 (22.6%)	6,748 (24.1%)	3,730 (20.4%)	<0.001
Chronic pain disorders	6,416 (13.9%)	3,796 (13.6%)	2,620 (14.3%)	0.028
Anxiety disorders	7,498 (16.2%)	4,391 (15.7%)	3,107 (17.0%)	<0.001
Cellulitis/soft tissue infection	13,862 (30.0%)	7,677 (27.5%)	6,185 (33.8%)	<0.001
Bacteremia/systemic infection	36,800 (79.5%)	22,344 (79.9%)	14,456 (78.9%)	0.009
Sepsis	13,674 (29.5%)	7,906 (28.3%)	5,768 (31.5%)	<0.001
GERD	9,030 (19.5%)	5,652 (20.2%)	3,378 (18.4%)	<0.001
Benign prostatic hyperplasia	2,960 (6.4%)	1,816 (6.5%)	1,144 (6.2%)	0.290
Osteoarthritis	7,278 (15.7%)	4,968 (17.8%)	2,310 (12.6%)	<0.001
Urinary tract infection	3,977 (8.6%)	2,210 (7.9%)	1,767 (9.6%)	<0.001

Data are presented as n (%). All variables compared using Pearson chi-square test. NIS = National Inpatient Sample; AKI = Acute kidney injury; COPD = Chronic obstructive pulmonary disease; GERD = Gastroesophageal reflux disease. Surgery defined by ICD-10-PCS primary procedure code (I10_PR1). p < 0.05 considered statistically significant.

In-hospital outcomes stratified by treatment group are presented in Table 3. Overall in-hospital mortality for septic joint admissions was 2.1%. Patients undergoing surgical management demonstrated significantly lower mortality compared with those treated non-surgically (1.4% vs. 3.1%, p < 0.001). The median length of stay for the overall cohort was seven days (IQR 4–12). While the median length of stay was similar between groups, the non-surgical cohort showed a wider interquartile range, reflecting longer hospitalizations (7 [4–13] days vs. 7 [4–11] days in the surgical group, p < 0.001). Total hospital charges also differed significantly between treatment groups. The overall median total charge was $68,822 (IQR $39,607–$124,019). Patients managed surgically had higher median hospital charges compared with non-surgically treated patients ($71,725 vs. $63,434, p < 0.001). Overall, surgical management was associated with lower in-hospital mortality but higher hospital charges, while non-surgical management was associated with slightly longer hospital stays.

**Table 3:** In-hospital outcomes stratified by treatment group. Septic joints, NIS Database 2016–2021 (known anatomic sites only)

Outcome	Overall (N = 46,282)	Surgery (n = 27,961)	No Surgery (n = 18,321)	*p*-value
In-hospital mortality, n (%)	952 (2.1%)	384 (1.4%)	568 (3.1%)	<0.001
Length of stay, median [IQR] (days)	7.0 [4.0–12.0]	7.0 [4.0–11.0]	7.0 [4.0–13.0]	<0.001
Total charges, median [IQR] (USD)	$68,822 [$39,607–$124,019]	$71,725 [$43,879–$123,910]	$63,434 [$32,574–$124,312]	<0.001

In-hospital mortality presented as n (%), compared using Pearson chi-square test. Length of stay and total hospital charges presented as median [interquartile range]; compared using Mann-Whitney U test. NIS = National Inpatient Sample; IQR = Interquartile range; USD = United States dollars. Surgery defined by ICD-10-PCS primary procedure code (I10_PR1). p < 0.05 considered statistically significant.

In-hospital complications stratified by treatment group are summarized in Table 4. Non-surgically managed patients experienced consistently higher complication rates. Sepsis or septic shock occurred in 29.5% overall and was more frequent in the non-surgical group (31.5% vs. 28.3%, p < 0.001), as was acute kidney injury (24.8% vs. 21.4%, p < 0.001). Respiratory and thromboembolic events were also increased, including acute respiratory failure (12.0% vs. 7.0%) and deep vein thrombosis or pulmonary embolism (8.9% vs. 5.8%) (both p < 0.001). Osteomyelitis was slightly more common in the non-surgical cohort (25.7% vs. 23.4%, p < 0.001). Cardiovascular complications, including myocardial infarction and stroke, occurred more frequently in non-surgical patients (2.5% vs. 1.4% and 1.5% vs. 0.8%, respectively; both p < 0.001). Hospital-acquired infections, including pneumonia (6.6% vs. 3.5%) and urinary tract infection (9.0% vs. 7.5%), as well as cardiac arrest (1.0% vs. 0.3%), were also more prevalent (all p < 0.001). Amputation rates were marginally higher (4.6% vs. 4.1%, p = 0.012). Overall, non-surgical management was associated with increased systemic and infectious morbidity.

**Table 4:** In-hospital complications among patients with septic joint admissions stratified by surgical versus non-surgical management

Complication	Overall (N = 46,282)	Surgery (n = 27,961)	No Surgery (n = 18,321)	*p*-value
Sepsis/Septic shock	13,674 (29.5%)	7,906 (28.3%)	5,768 (31.5%)	<0.001
Acute kidney injury (AKI)	10,536 (22.8%)	5,989 (21.4%)	4,547 (24.8%)	<0.001
Acute respiratory failure	4,160 (9.0%)	1,962 (7.0%)	2,198 (12.0%)	<0.001
DVT/Pulmonary embolism	3,250 (7.0%)	1,613 (5.8%)	1,637 (8.9%)	<0.001
Osteomyelitis	11,254 (24.3%)	6,552 (23.4%)	4,702 (25.7%)	<0.001
Acute myocardial infarction	852 (1.8%)	398 (1.4%)	454 (2.5%)	<0.001
Stroke/CVA	494 (1.1%)	213 (0.8%)	281 (1.5%)	<0.001
Pneumonia (hospital-acquired)	2,186 (4.7%)	971 (3.5%)	1,215 (6.6%)	<0.001
Urinary tract infection (hospital-acquired)	3,734 (8.1%)	2,086 (7.5%)	1,648 (9.0%)	<0.001
Cardiac arrest	266 (0.6%)	91 (0.3%)	175 (1.0%)	<0.001
Amputation	1,981 (4.3%)	1,143 (4.1%)	838 (4.6%)	0.012
Wound/Surgical site infection	11,207 (24.2%)	6,356 (22.7%)	4,851 (26.5%)	<0.001

Data are presented as n (%). All variables compared using Pearson chi-square test. NIS = National Inpatient Sample; AKI = Acute kidney injury; DVT = Deep vein thrombosis; CVA = Cerebrovascular accident. Complications identified from any ICD-10 diagnosis or procedure field. Surgery defined by ICD-10-PCS primary procedure code (I10_PR1). p < 0.05 considered statistically significant.

Following 1:1 propensity score matching, 18,258 patients remained in each treatment group (n = 36,516 total). Baseline demographic, hospital, and comorbidity characteristics were well balanced between cohorts, with all standardized mean differences below the accepted threshold of 0.10, indicating successful matching (Supplementary Table 1 & 2). Following the 1:1 propensity score matching as presented in Table 5, differences in several complications persisted between treatment groups. Patients managed non-surgically demonstrated significantly higher rates of acute respiratory failure (13.7% vs. 9.5%, p < 0.001), deep vein thrombosis or pulmonary embolism (8.1% vs. 5.6%, p < 0.001), acute myocardial infarction (1.5% vs. 0.8%, p < 0.001), stroke (1.4% vs. 0.8%, p < 0.001), and hospital-acquired pneumonia (12.9% vs. 8.5%, p < 0.001) compared with surgically treated patients. In contrast, rates of sepsis or septic shock, acute kidney injury, osteomyelitis, hospital-acquired urinary tract infection, cardiac arrest, and wound or surgical site infection were comparable between groups. Amputation was more frequently observed in the surgical cohort (11.6% vs. 3.7%, p < 0.001). Overall, after balancing baseline demographic and clinical characteristics, non-surgical management remained associated with a higher burden of several major systemic complications.

**Table 5:** In-hospital complications among patients with septic joint admissions stratified by surgical versus non-surgical management 1:1 Propensity Score Matching

Complication	Overall (N = 36,516)	Surgery (n = 18,258)	Non-Surgical (n = 18,258)	p-value
Sepsis/Septic shock	11,279 (30.9)	5,625 (30.8)	5,654 (31.0)	0.751
Acute kidney injury	9,040 (24.8)	4,523 (24.8)	4,517 (24.7)	0.952
Acute respiratory failure	4,231 (11.6)	1,729 (9.5)	2,502 (13.7)	<0.001
DVT/Pulmonary embolism	2,495 (6.8)	1,022 (5.6)	1,473 (8.1)	<0.001
Osteomyelitis	9,402 (25.7)	4,716 (25.8)	4,686 (25.7)	0.729
Acute myocardial infarction	419 (1.1)	148 (0.8)	271 (1.5)	<0.001
Stroke/CVA	415 (1.1)	153 (0.8)	262 (1.4)	<0.001
Hospital-acquired pneumonia	3,905 (10.7)	1,550 (8.5)	2,355 (12.9)	<0.001
Hospital-acquired urinary tract infection	3,540 (9.7)	1,734 (9.5)	1,806 (9.9)	0.209
Cardiac arrest	7,247 (19.8)	3,658 (20.0)	3,589 (19.7)	0.372
Amputation	2,793 (7.6)	2,119 (11.6)	674 (3.7)	<0.001
Wound/Surgical site infection	10,509 (28.8)	5,225 (28.6)	5,284 (28.9)	0.503

**Fig. 3:**
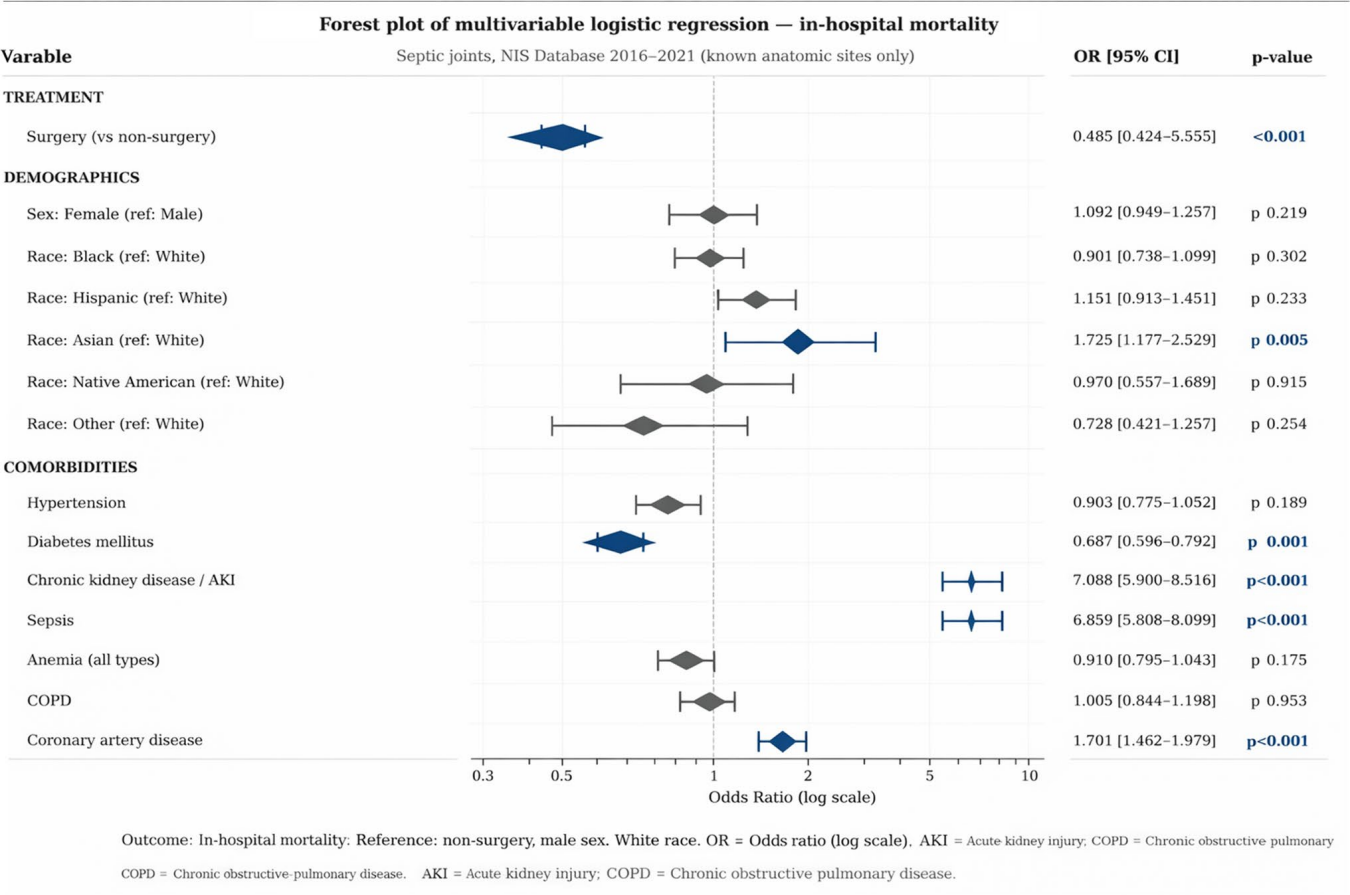
Forest plot of multivariable logistic regression for in-hospital mortality

Figure 3 presents adjusted odds ratios from the multivariable logistic regression analysis of in-hospital mortality. Surgical management was independently associated with significantly lower mortality compared with non-surgical treatment (OR 0.485, 95% CI 0.424–0.555, p < 0.001). Female sex was not associated with mortality. Compared with White patients, Asian race was associated with increased mortality risk, whereas other racial groups showed no significant differences. Comorbidities independently associated with increased mortality included chronic kidney disease/acute kidney injury, sepsis, and coronary artery disease. In contrast, diabetes mellitus was associated with lower mortality. Hypertension, anemia, and chronic obstructive pulmonary disease were not significant after adjustment. Overall, surgical management remained protective, while systemic illness and cardiovascular comorbidity were the strongest predictors of in-hospital mortality.

## Discussion

In this nationwide analysis of 46,282 hospitalizations for native septic arthritis with known anatomic sites, surgical management was performed in 60.4% of patients and was associated with significantly lower in-hospital mortality compared with non-surgical treatment (1.4% vs. 3.1%). Median length of stay was similar between groups; however, non-surgically managed patients demonstrated greater variability with more prolonged hospitalizations, whereas surgical treatment was associated with higher total hospital charges. Importantly, non-surgical management was consistently associated with higher rates of in-hospital complications, including sepsis, acute kidney injury, respiratory failure, thromboembolic events, and hospital-acquired infections. Multivariable analysis confirmed that surgical intervention remained independently associated with reduced odds of in-hospital mortality. In contrast, chronic kidney disease, sepsis, and coronary artery disease were strong independent predictors of mortality, whereas diabetes mellitus was associated with lower adjusted mortality. The knee was the most commonly affected joint, followed by the hip and ankle, with increasing case numbers from 2016 to 2019 and a decline during 2020–2021.

Importantly, the present cohort was restricted to native septic arthritis and did not include prosthetic joint infection. Therefore, these findings should be interpreted specifically within the context of native joint infection rather than arthroplasty-related infection, which involves distinct microbiologic characteristics, biofilm formation, and surgical treatment paradigms.

These findings are consistent with prior studies demonstrating improved outcomes with surgical drainage. A large cohort analysis reported significantly lower mortality among patients undergoing surgical debridement compared with medical management alone (14.7% vs 33.3%; adjusted OR 0.23, 95% CI 0.09–0.57). [20] Similarly, our results demonstrate both reduced mortality and lower rates of systemic complications among surgically treated patients, supporting a potential association between surgical source control and improved clinical outcomes in septic arthritis.

Nevertheless, the comparative evidence remains heterogeneous. While some studies suggest that serial needle aspiration may achieve comparable outcomes in selected patients, approximately 30% of medically managed cases ultimately require surgical intervention, with rates approaching 50% in shoulder and hip infections. [21] A single-center UK study reported no significant differences in recovery or mortality between medical and surgical treatment strategies. [12] These findings should be interpreted cautiously, as non-operative cohorts frequently include patients with greater comorbidity burden and illness severity. In our study, non-surgically managed patients exhibited higher rates of chronic kidney disease, coronary artery disease, anemia, and active infection, suggesting confounding by indication despite adjustment.

The timing of intervention appears to be a critical determinant of outcomes. A multicenter study demonstrated that delayed surgical treatment was associated with increased one year mortality (HR 1.7, 95% CI 1.1–2.6) and treatment failure (HR 1.5, 95% CI 1.1–2.0). [22] Additional analyses indicate that each day of delay confers a substantial increase in adverse outcomes. [22] In our cohort, systemic illness markers—including sepsis and major comorbidities—were strongly associated with mortality, suggesting that earlier surgical source control may be associated with improved outcomes before clinical deterioration occurs.

However, evidence regarding optimal timing remains inconsistent. A Swiss cohort study of 204 patients found no association between timing of drainage and long-term sequelae, with similar outcomes regardless of whether lavage was performed within six hours or after 24 hours. [23] This variability likely reflects differences in disease severity, joint involvement, and host factors, emphasizing the need for individualized clinical decision-making.

Several predictors of treatment failure have been described, including prolonged symptom duration, inflammatory arthropathy (OR 7.3), large joint involvement (OR 7.0), elevated synovial leukocyte count (>85 × 10⁹ cells/L; OR 4.7), *Staphylococcus aureus* infection (OR 4.6), and diabetes mellitus (OR 2.6). [24] Notably, up to 38% of patients require more than one surgical debridement, reflecting the complexity of infection control. Poor functional outcomes including amputation, arthrodesis, or prosthetic reconstruction occur in 24–33% of cases. [21] These data, together with our findings of higher complication rates in non-surgically treated patients, reinforce the importance of timely and appropriate intervention.

Economic considerations also influence treatment decisions. Some studies suggest that medical management may reduce short-term healthcare utilization, including lower rehabilitation requirements, although this may be offset by longer hospital stays and increased complications. [12, 25] Non-operative treatment has been associated with lower discharge to rehabilitation and fewer readmissions, but higher short-term mortality, likely reflecting patient selection. [26] In contrast, our nationwide analysis demonstrated that surgical management was associated with higher hospital charges but significantly improved clinical outcomes. These findings suggest that although non-surgical strategies may reduce short-term costs, surgical management was associated with improved short-term clinical outcomes in appropriately selected patients. [2]

Chronic kidney disease, cardiovascular disease, and systemic sepsis are well-established predictors of mortality in septic arthritis. Reported mortality rates range from 7–18% at 90 days and 17–28% at one to five years. [27–29] These comorbidities likely contribute through impaired immune function and reduced physiologic reserve. [27, 28, 30, 31] Similarly, systemic sepsis and bacteraemia are strongly associated with worse outcomes. [31–33] In contrast, diabetes mellitus demonstrated a paradoxical association with lower adjusted mortality in our analysis, a finding previously reported but not fully understood. [28, 34, 35] Overall, mortality appears to be driven predominantly by systemic illness severity rather than infection alone.

This study has several limitations. The use of an administrative database introduces potential misclassification due to reliance on ICD-10 coding. Despite efforts to exclude prosthetic joint infection using ICD-10 coding, misclassification remains possible because of inherent limitations of administrative datasets. Important clinical variables including laboratory parameters, microbiology, symptom duration, imaging findings, and timing of intervention are unavailable, limiting risk adjustment. Treatment allocation was not randomized, and non-surgical patients had a higher burden of comorbidities, introducing confounding by indication. Furthermore, the database captures only in-hospital outcomes and lacks information on long-term complications, functional outcomes, and readmissions. Procedural details, including surgical technique, cannot be reliably distinguished, and exclusion of cases with unspecified joint location may limit generalizability. The non-surgical cohort likely represented a heterogeneous population that may have included patients treated with antibiotic therapy alone, serial aspiration, bedside drainage procedures, delayed surgical intervention, or palliative treatment strategies. Because the NIS lacks detailed clinical management data, these treatment pathways could not be distinguished, which may limit the interpretation and clinical applicability of comparisons between treatment groups. Finally, the present study evaluated septic arthritis as a combined cohort and did not perform joint-specific subgroup analyses. As treatment strategies, patient characteristics, and outcomes may vary according to the affected anatomical site, future studies focusing on individual joints (e.g., knee, hip, and shoulder) are warranted to provide more granular clinical insights.

Despite these limitations, this study leverages a large, nationally representative dataset, providing robust real-world insight into the management of septic arthritis across diverse clinical settings. The substantial sample size allows for adequate statistical power, while inclusion of multiple joint sites enhances generalizability. Survey-weighted analyses strengthen the validity of national estimates, and multivariable adjustment improves the robustness of observed associations.

## Conclusions

In conclusion, surgical management of septic arthritis was associated with significantly lower in-hospital mortality and fewer systemic complications compared with non-surgical treatment, despite higher hospital charges. Chronic kidney disease, sepsis, and cardiovascular disease were strong predictors of mortality, highlighting the impact of systemic illness. These findings demonstrate an association between surgical management and improved in-hospital outcomes among appropriately selected patients; however, causality cannot be established, and prospective studies are needed to better define the relationship between treatment strategy and clinical outcomes. Prospective studies are warranted to better define optimal patient selection, timing of intervention, and long-term outcomes.

## Supplementary Information

Below is the link to the electronic supplementary material.

Supplementary file1 (DOCX 24 KB)

## Data Availability

This study utilized previously collected, de-identified research data from the National Inpatient Sample (2016–2021), which were analyzed for the purposes of this investigation. No new primary data were generated.
