# Supplementary material for: Management of septic joints in the United States: A Nationwide comparison of surgical and non-surgical treatment
Source: Int Orthop. 2026 Jun 24;50(7):1523–34. doi: 10.1007/s00264-026-06909-w (PMC13407719; doi:10.1007/s00264-026-06909-w)
Supplement: Supplementary file 1 — Supplementary file1 (DOCX 24 KB) [file 264_2026_6909_MOESM1_ESM.docx]

1. **Supplementary Material:**

### **Table 1s. Baseline Demographic and Hospital Characteristics After 1:1 Propensity Score Matching**

| Characteristic | Overall (N = 36,516) | Surgery (n = 18,258) | Non-Surgical (n = 18,258) | SMD |
| --- | --- | --- | --- | --- |
| **Sex** |  |  |  |  |
| Male | 23,164 (63.4) | 11,582 (63.4) | 11,582 (63.4) | 0.000 |
| Female | 13,352 (36.6) | 6,676 (36.6) | 6,676 (36.6) | 0.000 |
| **Race/Ethnicity** |  |  |  |  |
| White | 25,395 (69.5) | 12,742 (69.8) | 12,653 (69.3) | 0.011 |
| Black | 4,641 (12.7) | 2,312 (12.7) | 2,329 (12.8) | 0.003 |
| Hispanic | 3,283 (9.0) | 1,645 (9.0) | 1,638 (9.0) | 0.001 |
| Asian/Pacific Islander | 628 (1.7) | 322 (1.8) | 306 (1.7) | 0.008 |
| Native American | 540 (1.5) | 268 (1.5) | 272 (1.5) | 0.002 |
| Other | 787 (2.2) | 388 (2.1) | 399 (2.2) | 0.007 |
| **Primary Payer** |  |  |  |  |
| Medicare | 16,964 (46.5) | 8,450 (46.3) | 8,514 (46.6) | 0.006 |
| Medicaid | 7,908 (21.7) | 3,773 (20.7) | 4,135 (22.6) | 0.047 |
| Private Insurance | 8,359 (22.9) | 4,446 (24.4) | 3,913 (21.4) | 0.071 |
| Self-Pay | 1,794 (4.9) | 851 (4.7) | 943 (5.2) | 0.024 |
| No Charge | 167 (0.5) | 80 (0.4) | 87 (0.5) | 0.011 |
| Other | 1,260 (3.5) | 631 (3.5) | 629 (3.4) | 0.002 |
| **Hospital Bed Size** |  |  |  |  |
| Small | 7,573 (20.7) | 3,803 (20.8) | 3,770 (20.6) | 0.005 |
| Medium | 9,849 (27.0) | 4,999 (27.4) | 4,850 (26.6) | 0.018 |
| Large | 19,094 (52.3) | 9,456 (51.8) | 9,638 (52.8) | 0.020 |

*Table 1s. Data are presented as n (%). Balance between matched cohorts was assessed using standardized mean differences (SMDs). An SMD <0.10 was considered indicative of adequate covariate balance. Propensity score matching was performed using 1:1 nearest-neighbor matching without replacement. N = number of patients.*

**Table 2s. Baseline Comorbidities After 1:1 Propensity Score Matching**

| Comorbidity | Overall (N = 36,516) | Surgery (n = 18,258) | Non-Surgical (n = 18,258) | SMD |
| --- | --- | --- | --- | --- |
| Hypertension | 21,721 (59.5) | 10,858 (59.5) | 10,863 (59.5) | 0.001 |
| Hyperlipidemia | 11,104 (30.4) | 5,526 (30.3) | 5,578 (30.6) | 0.006 |
| Smoking/Nicotine use | 14,404 (39.4) | 7,197 (39.4) | 7,207 (39.5) | 0.002 |
| Diabetes mellitus | 13,299 (36.4) | 6,673 (36.5) | 6,626 (36.3) | 0.004 |
| Coronary artery disease | 6,685 (18.3) | 3,322 (18.2) | 3,363 (18.4) | 0.005 |
| Chronic kidney disease/AKI | 13,231 (36.2) | 6,575 (36.0) | 6,656 (36.5) | 0.010 |
| Anemia (all types) | 16,699 (45.7) | 8,349 (45.7) | 8,350 (45.7) | <0.001 |
| Electrolyte disorders | 13,757 (37.7) | 6,865 (37.6) | 6,892 (37.7) | 0.002 |
| Hypothyroidism | 3,943 (10.8) | 1,984 (10.9) | 1,959 (10.7) | 0.006 |
| COPD | 6,518 (17.8) | 3,250 (17.8) | 3,268 (17.9) | 0.002 |
| Obesity/Morbid obesity | 7,467 (20.4) | 3,740 (20.5) | 3,727 (20.4) | 0.003 |
| Chronic pain disorders | 5,208 (14.3) | 2,601 (14.2) | 2,607 (14.3) | 0.001 |
| Anxiety disorders | 6,178 (16.9) | 3,087 (16.9) | 3,091 (16.9) | <0.001 |
| Cellulitis/Soft tissue infection | 12,326 (33.8) | 6,190 (33.9) | 6,136 (33.6) | 0.006 |
| Bacteremia/Systemic infection | 28,786 (78.8) | 14,385 (78.8) | 14,401 (78.9) | 0.002 |
| Sepsis | 11,410 (31.2) | 5,684 (31.1) | 5,726 (31.4) | 0.006 |
| GERD | 6,802 (18.6) | 3,428 (18.8) | 3,374 (18.5) | 0.008 |
| Benign prostatic hyperplasia | 2,268 (6.2) | 1,129 (6.2) | 1,139 (6.2) | 0.002 |
| Osteoarthritis | 4,645 (12.7) | 2,336 (12.8) | 2,309 (12.6) | 0.006 |
| Urinary tract infection | 3,434 (9.4) | 1,702 (9.3) | 1,732 (9.5) | 0.007 |

*Table 2s. Data are presented as n (%). Balance between matched groups was assessed using standardized mean differences (SMDs). An SMD < 0.10 was considered indicative of adequate covariate balance. COPD = chronic obstructive pulmonary disease; AKI = acute kidney injury; GERD = gastroesophageal reflux disease.*
